# Molecular Imaging in Endometrial Cancer: A Narrative Review

**DOI:** 10.3390/cancers17162608

**Published:** 2025-08-08

**Authors:** Ana María García-Vicente, María Pilar Perlaza-Jiménez, Stefanía Aida Guzmán-Ortiz, Marta Tormo-Ratera, Ana Sánchez-Márquez, Montserrat Cortés-Romera, Edel Noriega-Álvarez

**Affiliations:** 1 Nuclear Medicine Department, University Hospital of Toledo, 45007 Toledo, Spain; stefaniag@sescam.jccm.es; 2 Nuclear Medicine-PET (ICS-IDI) Department, Bellvitge University Hospital, L’Hospitalet de Llobregat, 08907 Barcelona, Spain; mariadelpilar.perlaza.idi@gencat.cat (M.P.P.-J.); martatormo.idi@gencat.cat (M.T.-R.); mcortes@bellvitgehospital.cat (M.C.-R.); 3 Radiology Department, Bellvitge University Hospital, L’Hospitalet de Llobregat, 08907 Barcelona, Spain; ana.sanchez.idi@gencat.cat; 4 Department of Nuclear Medicine, University Hospital of Guadalajara, 19002 Guadalajara, Spain; edelnoriega@gmail.com

**Keywords:** endometrial cancer, [^18^F]FDG-PET/CT, [^18^F]FDG-PET/MRI, radiomics, staging, response, progression, prognosis

## Abstract

**Background/Objectives**: Endometrial cancer (EC) is the most common gynecological tumor in developed countries, and presents a wide variety of histological and molecular characteristics that make its treatment increasingly complex. In recent years, advances in molecular imaging, particularly with [^18^F]FDG-PET/CT and PET/MRI, have changed clinicians’ management of diagnosis, treatment planning, and prognosis of EC. **Methods**: In this narrative review, a search was conducted for current evidence on the role of [^18^F]FDG-PET/CT and PET/MRI throughout the treatment of EC, focusing on their diagnostic performance, clinical relevance, and prognostic implications. Their use in areas such as initial staging, treatment monitoring, recurrence detection, and individualized risk assessment was also discussed. **Results**: [^18^F]FDG-PET/CT is effective in detecting lymph node and distant metastases, while [^18^F]FDG-PET/MRI offers greater accuracy for T and N staging. In addition, [^18^F]FDG-PET/CT provides early metabolic indicators of therapeutic response and facilitates differentiation between viable tumors and post-treatment changes. Radiomics-derived parameters have shown promising associations with tumor aggressiveness and lymphovascular invasion and survival, supporting their role as prognostic imaging biomarkers. In addition, the use of non-FDG radiotracers, as well as predictive models based on machine learning, can further refine preoperative stratification and treatment planning in certain subtypes of EC. **Conclusions**: Molecular imaging enhances the accuracy of TNM staging in EC. The incorporation of molecular imaging biomarkers into daily clinical practice could significantly improve personalized decision-making in EC. However, large prospective studies are needed to confirm their efficacy.

## Simple Summary

Endometrial cancer is the most common cancer of the female reproductive system in developed countries. Its diagnosis and treatment can be difficult due to the wide variety of tumor types and behaviors. This narrative review explores how diagnostic imaging techniques, particularly molecular imaging methods such as PET/CT and PET/MRI, can help clinicians better understand and manage this disease. These techniques not only improve the detection of tumor spread and response to treatment, but also provide biological information about the cancer in each patient. This knowledge allows for more personalized and effective therapeutic decision-making. The authors also discuss new imaging methods and artificial intelligence-assisted models that could further refine risk assessment in the future. This work highlights the important role of advanced imaging techniques in improving outcomes for women with endometrial cancer, and encourages further research to confirm these promising benefits.

## 1. Introduction

Endometrial cancer (EC) is the most common malignancy of the female reproductive system in developed countries. Histopathologically, two main subtypes have been described: type I tumors (>80%), which are estrogen-dependent (and include grade 1 and 2 endometrioid adenocarcinomas), and type II tumors, which are not estrogen-dependent (include the more clinically aggressive grade 3 endometrioid adenocarcinomas, serous papillary adenocarcinomas, clear cell adenocarcinomas, and carcinosarcomas) [1]. Type II tumors demonstrate a worse prognosis and are responsible for nearly half of the EC-related deaths [2].

**Figure 1:**
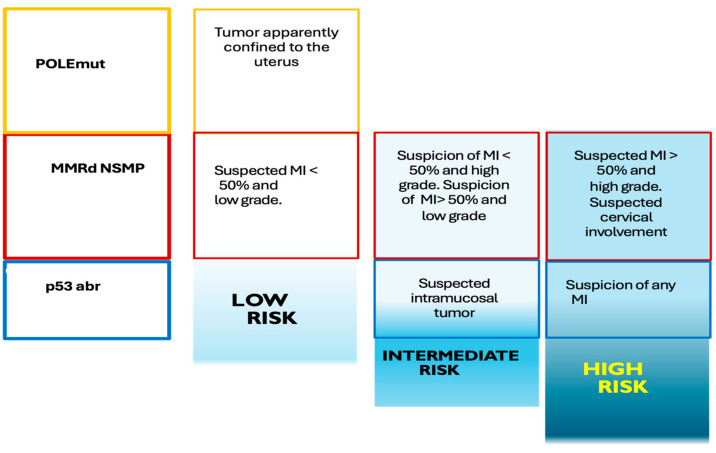
Preoperative risk groups apparently confined to the uterus with molecular classification. MI: myometrial invasion. POLEmut: Molecular subgroup with mutation in the *POLE* gene. MMRd: Molecular subgroup with mutation in DNA repair proteins. NSMP: Molecular subgroup that does not have mutation in POLE, p53, or DNA repair proteins. p53abr: Molecular subgroup with mutation in p53.

The 2016 European Society of Gynecological Oncology (ESGO) and the European Society for Radiotherapy and Oncology (ESTRO) consensus defined the high-risk group as endometrioid carcinomas of stage IB with a deep myometrial invasion (MI) > 50% grade 3 or stage II–III any grade along with non-endometrioid carcinomas of stage I–III [3,4]. However, a molecular classification recommended by ESGO, ESTRO, and European Society of Pathology guidelines has been fully integrated in the clinical routine for the risk classification of EC patients [3,4,5,6]. Figure 1 represents an adaptation of the molecular classification of EC.

The loss of expression of DNA repair proteins (MLH1, PMS2, MSH2, MSH6) reflects a high probability that the tumor presents high microsatellite instability, which leads to an intermediate prognosis. Similarly, an abnormal pattern of p53 staining is associated with alterations (mutations) in the *TP53* gene, which confers a worse prognosis, while the detection of mutations in the *POLE* gene is associated with tumors with a good prognosis with a lower risk of recurrence. Adaptation from SEGO (Spanish Society of Ginecological Oncology) Oncoguide: Endometrial cancer 2023. ISBN: 978-84-09-40278-6.

The molecular characterization of EC increases the accuracy of the risk classification solely based on histological subtype, grade, and MI [5]. Thus, when molecular classification reveals an aberrant p53 or *POLE* mutation in stages I and II, this results in overstaging or downstaging of the disease, respectively [3]. Molecular subtypes independently predict clinical outcomes, helping inform risk stratification, particularly when considering whether to utilize adjuvant chemoradiotherapy (CRT) [6].

Most patients with EC are confined to the uterine corpus at the time of diagnosis, with early stage and good prognosis. However, approximately 10–15% of patients with EC present with advanced-stage and harbor disease beyond the uterus and abdominopelvic lymph nodes [7]. Although the International Federation of Gynecology and Obstetrics (FIGO) staging system is primarily surgical, complementary presurgical imaging adds a layer of precision to evaluate tumor size, myometrial and cervical involvement, adnexal status, and lymph node involvement [8]. Therefore, lymphadenectomy, for staging purposes, is usually reserved for patients with high-risk disease because of its associated perioperative complications and long-term morbidity. Table 1 summarizes FIGO stage current classification.

Positron emission tomography/computed tomography (PET/CT) and positron emission tomography/magnetic resonance imaging (PET/MRI), with fluorine-18-2-fluoro-2-deoxy-D-glucose ([^18^F]FDG), are diagnostic techniques with high specificity and positive predictive value (PPV) in gynecological tumor staging, treatment response, recurrence detection, and prognosis prediction.

The following sections present a review of the current state of PET/CT and PET/MRI using [^18^F]FDG and others radiotracers in EC.

## 2. Initial Staging

### 2.1. T Stage

Accurate assessment of the primary tumor (T) stage, encompassing the depth of MI and potential extension to adjacent structures such as the cervix, is fundamental for therapeutic planning in EC. Scientific evidence indicates that [^18^F]FDG-PET/CT is a technique that can facilitate improved diagnosis and, consequently, treatment of EC. Currently, [^18^F]FDG-PET/CT is used as an adjunct to conventional diagnostic imaging techniques. This is due to its limited spatial resolution, which hinders the precise assessment of small tumors and the exact delineation of local invasion; for this reason, the diagnostic performance of [^18^F]FDG-PET/CT for the primary tumor is inferior to that of MRI. Therefore, [^18^F]FDG-PET/CT is not recommended for screening or early diagnosis of primary lesions [9]. Thus, for assessing the T stage of EC, MRI and, in certain contexts, transvaginal ultrasound performed by experts are recommended, as these modalities offer superior performance for these purposes [9,10].

Based on current evidence, [^18^F]FDG-PET/CT is not considered the imaging modality of choice for the detailed evaluation of T staging. However, although [^18^F]FDG-PET/CT does not replace MRI for T stage determination, the metabolic information it provides could add additional biological characterization [11,12,13,14]. This implies providing functional information that could influence the interpretation of preoperative risk and, potentially, clinical decision-making.

[^18^F]FDG-PET/MRI has demonstrated high accuracy in the preoperative evaluation of EC, particularly in determining deep MI, with values around 80%, outperforming [^18^F]FDG-PET/CT [15,16,17,18]. Similarly, in a study by Yu et al. [17], [^18^F]FDG-PET/MRI showed a sensitivity, specificity, and accuracy of 89%, 95%, and 93%, respectively, for detecting MI in a cohort of 57 patients with EC. Other study reported an accurate detection of cervical invasion with a sensitivity, specificity, and accuracy of 81%, 95%, and 91%, respectively. Additionally, quantitative parameters extracted from PET and MRI, as SUVmax/minimum apparent diffusion coefficient (ADCmin) index, showed association with clinicopathological characteristics of aggressiveness [17,19].

### 2.2. N Stage

The detection of regional lymph node metastases (N stage) is a prognostic factor of great importance in EC and has direct implications for treatment planning.

Recent clinical studies support the use of [^18^F]FDG-PET/CT for nodal staging, as it possesses greater reliability in diagnosing pelvic and para-aortic lymph node metastases, compared to conventional imaging modalities such as CT or MRI alone [9,20].

Clinical guidelines such as those from the European Society for Medical Oncology (ESMO) indicate that [^18^F]FDG-PET/CT can be considered as an additional diagnostic test in the high-risk patient group to detect preoperative nodal extension in patients with EC [10]. Likewise, the clinical guidelines of the National Comprehensive Cancer Network (NCCN) and the Spanish Society of Medical Oncology, in collaboration with the Spanish Group for Ovarian Cancer Research (SEOM-GEICO), recommend that [^18^F]FDG-PET/CT should be considered if metastases are suspected, in selected cases in patients eligible for surgery or locoregional therapy [21,22]. The European Association of Nuclear Medicine (EANM) and the Society of Nuclear Medicine and Molecular Imaging (SNMMI) have not specific guidelines dedicated to the evaluation of EC, although for cervical cancer, they highlight that CT and MRI are used for the evaluation of locoregional lymph node involvement. However, [^18^F]FDG-PET/CT exhibits higher sensitivity for the detection of metastases, including para-aortic nodal involvement, compared to MRI limited to the pelvis. Thus, this diagnostic approach could also be considered applicable, in certain clinical contexts, to the management of EC [23].

A retrospective study that included patients with clinical stage I based on MRI and [^18^F]FDG-PET/CT detected lymph node metastases at both pelvic and para-aortic levels in high-risk patients, as well as a higher recurrence rate. It is noteworthy that in this study, even patients in the low/intermediate-risk group presented lymph node metastases and nodal recurrence. This dual imaging assessment had a negative predictive value (NPV) of 0.945, although the low prevalence of lymph node involvement (5.5%) could bias the results. Therefore, it would be interesting to conduct prospective studies to confirm these findings, as it could help select a subgroup of low-risk patients in whom omitting complete surgical lymphadenectomy would lead to a significant reduction in the morbidity associated with these procedures, such as lymphedema. However, this approach requires robust validation and careful consideration of the inherent risk of not detecting micrometastases, which currently escapes the resolution of [^18^F]FDG-PET/CT [24].

Corroborating the aforementioned information, Fasmer et al. [14] evaluated a dual imaging approach using MRI in all patients and [^18^F]FDG-PET/CT only in selected cases based on MRI findings, which allowed the identification of preoperative risk groups with significant differences in survival. The approach selected approximately 54% of patients for [^18^F]FDG-PET/CT, demonstrating an improvement in the detection of lymph node metastases compared to MRI alone. Likewise, they demonstrated that imaging using MRI and [^18^F]FDG-PET/CT offers comparable diagnostic performance in low and high histological risk groups for predicting central parameters of the FIGO stage [25].

[^18^F]FDG-PET/CT has demonstrated moderate sensitivity and high specificity for the detection of pelvic lymph node metastases. The sentinel lymph node (SLN) mapping technique along with thorough histopathological staging increased the identification of micrometastases in pelvic lymph nodes (incidence from 18.3% to 27.3%), which are not detectable by [^18^F]FDG-PET/CT due to its limited spatial resolution. That is, [^18^F]FDG-PET/CT may not detect micrometastases, which can be identified by thorough histopathological staging applied to surgically obtained sentinel nodes. Therefore, the combination of both modalities is promising for nodal staging purposes [13,26].

Fasmer et al. [13] demonstrated that, although [^18^F]FDG-PET/CT has limitations in diagnosing micrometastases due to its reduced spatial resolution, an elevated metabolic tumor volume (MTV) could be associated with a higher risk of micrometastases, as it showed high diagnostic performance for predicting lymph node metastases and aggressive disease, representing a promising complement to conventional [^18^F]FDG-PET/CT interpretation.

Previous studies comparing [^18^F]FDG-PET/MRI and [^18^F]FDG-PET/CT for N staging have reported contradictory results with comparable performance for both [18] vs. higher accuracies for [^18^F]FDG-PET/MRI [15,16].

**Figure 2:**
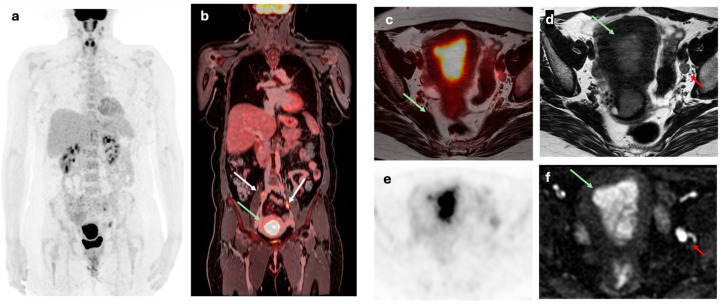
A 50-year-old woman with a FIGO IIIC1 EC. (**a**) Maximum intensity projection (MIP) and (**b**) coronal fused [^18^F]F-FDG PET/MRI staging images showing primary endometrial tumor with bilateral common iliac lymph node involvement (white arrows) and left external iliac node metastasis. (**c**) Axial fused [^18^F]F-FDG PET/MRI, (**d**) axial T2-weighted, (**e**) axial [^18^F]F-FDG PET, and (**f**) DWI images showing a heterogeneous on T2 with diffusion restriction of primary tumor (green arrow) and a 9 mm left external iliac lymph node with pathological FDG uptake (red arrow).

As disease extension is often unclear before surgery, Weissinger et al. [27] published a prospective study to evaluate [^18^F]FDG-PET/MRI as a minimal invasive technique to assess N staging. [^18^F]FDG-PET/MRI demonstrated high specificity in detecting para-aortic lymph nodes (99%), but moderate sensitivity (67%). The results were comparable in terms of pelvic lymph nodes, with a specificity of 98% and a sensitivity of 73%. Other authors support the excellent results of [^18^F]FDG-PET/MRI for N staging, showing high accuracy in detecting lymph node involvement (sensitivity: 86%, specificity: 93%, accuracy: 91%), making it especially effective for nodal staging [16]. Figure 2.

### 2.3. M Stage

The identification of distant metastases (M stage) at the time of initial diagnosis is a crucial prognostic factor that significantly alters the therapeutic approach, generally orienting it towards systemic treatments.

[^18^F]FDG-PET/CT is considered the most sensitive and useful non-invasive imaging modality for the detection of distant metastases in patients with EC, especially in high-risk cases or those with aggressive histological subtypes (clear cell/serous/dedifferentiated carcinomas and carcinosarcoma), surpassing CT and MRI in the detection of extraregional metastatic disease. Its early use in the diagnosis of high-risk patients could optimize the selection of candidates for cytoreductive surgery versus primary systemic therapy, avoiding unnecessary surgeries in patients with disseminated disease [28].

The ESMO clinical guideline suggests considering [^18^F]FDG-PET/CT as an additional diagnostic test in high-risk patients to detect preoperative extrapelvic extension and postoperative recurrence. In addition, a chest CT is recommended as part of the initial evaluation to rule out pulmonary metastases in patients with high-risk EC [10]. The NCCN clinical guideline recommends considering whole-body CT to assess the presence of metastatic disease in patients with high-grade carcinomas, in those with an incidental finding of cancer or incomplete staging who present significant risk factors, or if there is a well-founded clinical suspicion of metastases or in the context of suspected recurrence or subsequently developed metastatic disease. In addition, this guideline states that [^18^F]FDG-PET/CT can be considered in selected patients with suspected metastases [21,29]. The SEOM-GEICO clinical guideline indicates that a whole-body CT should be performed to rule out distant metastases, and [^18^F]FDG-PET/CT could also be used for this purpose in selected patients eligible for surgery or locoregional therapy [22].

**Figure 3:**
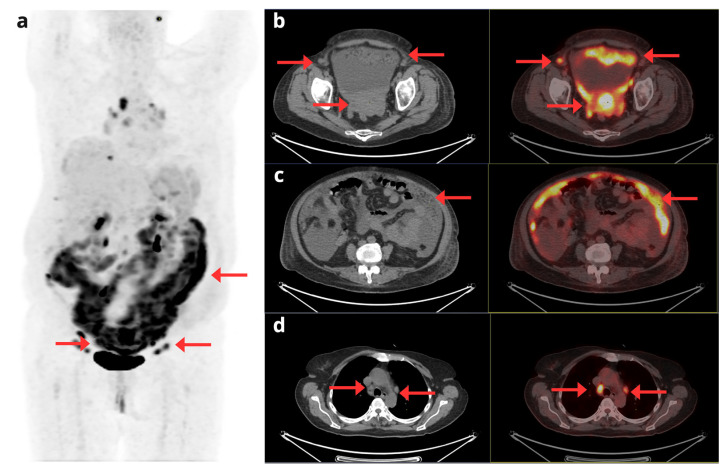
A 70-year-old woman with a diagnosis of stage IV serous EC. The maximum intensity projection PET image (**a**) shows extensive disease with uterine involvement, supra- and infradiaphragmatic adenopathy, and peritoneal involvement (red arrows). Fused axial CT and [^18^F]F-FDG PET/CT slices (**b**–**d**) show foci of pathologic hypermetabolism in the uterus, pelvic lymph nodes, and peritoneum (**b**); peritoneal implants in the greater omentum (**c**); and mediastinal adenopathy in the right paratracheal region and the aortopulmonary window (**d**).

As previously mentioned, although there is no specific SNMMI or EANM guideline for EC, the joint EANM/SNMMI guideline for cervical cancer mentions a higher sensitivity of [^18^F]FDG-PET/CT for the detection of metastases, an approach that could also be considered applicable in certain clinical contexts of EC [23]. For example, peritoneal dissemination has been observed in 10–20% of patients with high histological risk [30]. Likewise, Hong et al. [31] demonstrated that [^18^F]FDG-PET/CT significantly identified bone metastases in patients with type II endometrial carcinoma, especially with serous histology, which is associated with aggressive biology and poorer prognosis. Furthermore, they noted that [^18^F]FDG-PET/CT is superior to CT for detecting bone metastases and other non-visible distant metastases. Therefore, in these high-risk patients, [^18^F]FDG-PET/CT seems to be justified. Figure 3.

Data published in the literature define that [^18^F]FDG-PET/MRI has a high specificity and accuracy in assessing metastatic disease [19]. A retrospective study reported a sensitivity, specificity, and accuracy of M staging of 33%, 100%, and 82%, respectively, for [^18^F]FDG-PET/MRI, despite that in this study, [^18^F]FDG-PET/MRI was realized without gadolinium-based contrast [32]. In a prospective study including 35 patients with pathologically proven EC, [^18^F]FDG-PET/MRI revealed hepatic metastasis in one patient, which was histologically confirmed by biopsy [27]. Beyond its diagnostic performance, [^18^F]FDG-PET/MRI presents several additional advantages that enhance its clinical value. The combination of PET and MRI adds unique benefits when analyzing endometrial and menstrual changes, particularly in reproductive-age women and complex staging cases. Moreover, [^18^F]FDG-PET/MRI represents a viable alternative to conventional imaging methods, especially for patients who are unable to receive iodinated or gadolinium-based contrast agents. Another benefit is the lower radiation exposure associated with [^18^F]FDG-PET/MRI compared to [^18^F]FDG-PET/CT or CT imaging [19]. However, PET/MRI is a less available and more expensive technique. Table 2 summarizes the advantages and disadvantages, for diagnosis and staging, of both hybrid diagnostic techniques.

## 3. Image-Guided Surgery and Radiotherapy

Gynecological cancers have particularly benefitted from the increasing use of imaging to guide both surgery and radiation treatment planning [25,33,34]. In locally advanced EC, CRT is routinely administered as definitive therapy and can be used alone or in combination with surgery in the remaining stages [35]. The recent identification of molecular EC subtypes has led to the development of targeted therapies, particularly for early recurrent EC subtypes [36].

Although imaging is not a part of the staging system for EC, it does influence treatment selection [37,38]. MRI is the preferred imaging modality for preoperative assessment of depth of MI, cervical stromal involvement, extrauterine disease, and nodal involvement. In cases of local recurrence with invasion of adjacent organs or the pelvic sidewall, MRI should be used to assess the likelihood of surgical resectability and to plan salvage therapy. Moreover, [^18^F]FDG-PET/CT is helpful to exclude nodal and distant metastases, offering a greater accuracy than CT or MRI alone, especially for the evaluation of para-aortic lymph nodes [12]. In addition, the high positive predictive value (PPV) of [^18^F]FDG-PET/CT is useful for referring patients to appropriate cytoreductive surgery [12]. However, its main limitation lies in the detection of micrometastases or involvement in normal-sized lymph nodes, situations in which SLN mapping followed by exhaustive histopathological analysis may offer superior performance. Furthermore, [^18^F]FDG-PET/CT can be particularly useful in patients classified as high-risk to identify nodal disease not suspected by other techniques, which may influence the extent of lymphadenectomy to be performed or the planning of adjuvant radiotherapy. Therefore, the optimization of N staging likely resides in the combination of metabolic-anatomical information from [^18^F]FDG-PET/CT with the biological profile of the tumor. The issue of whether [^18^F]FDG-PET/CT or PET/MRI may reduce the need for systematic lymphadenectomy in low-risk patients by improving nodal assessment needs to be demonstrated in prospective studies.

## 4. Response to Treatment

### 4.1. Locoregional Disease

Following the initiation of therapy, monitoring tumor response is critical for guiding further clinical management. Unlike anatomical imaging, [^18^F]FDG-PET/CT enables functional assessment in EC by detecting early changes in glucose metabolism that typically precede morphological alterations. These metabolic changes may serve as early indicators of therapeutic efficacy, providing valuable information on tumor biology before the structural response becomes evident [39]. However, the clinical use of [^18^F]FDG-PET/CT in the evaluation of locoregional response to treatment in EC remains a developing field.

Viable tumor is more likely to show hypermetabolism and can therefore be differentiated from post-therapeutic changes on [^18^F]FDG-PET/CT. A key challenge lies in determining the optimal timing of post-therapy imaging, as metabolic changes caused by chemotherapy or radiotherapy—particularly inflammation—can result in false positive findings. Current evidence suggests that [^18^F]FDG-PET/CT should generally be delayed for 8–12 weeks after radiotherapy and at least 6 weeks after surgery to minimize interpretation errors. However, inflammatory changes may persist and interfere with interpretation even up to 6 months post-treatment in some cases. Despite these limitations, post-radiation changes seem to play a minimal impact of on [^18^F]FDG-PET’s diagnostic accuracy [40,41].

Few studies have investigated the utility of [^18^F]FDG-PET/CT in the assessment of EC response [42,43]. In one cohort of 21 patients undergoing [^18^F]FDG-PET/CT before and after chemotherapy, significant changes in [^18^F]FDG uptake were strongly correlated with treatment response, achieving a sensitivity of 90% and a specificity of 80% in distinguishing responders from non-responders. However, the relatively small cohort size and the moderate rate of false positives highlight the need for further validation in larger prospective studies [42]. Moreover, the degree of metabolic response demonstrated on [^18^F]FDG-PET/CT may carry prognostic value [43].

On the other hand, although [^18^F]FDG-PET/MRI is a promising imaging tool for monitoring treatment response in EC, especially in primary tumor, it has to be validated in order for international societies to reach consensus among the use of hybrid diagnostic techniques in this setting [3,4,5].

### 4.2. Distant Metastatic Disease

**Figure 4:**
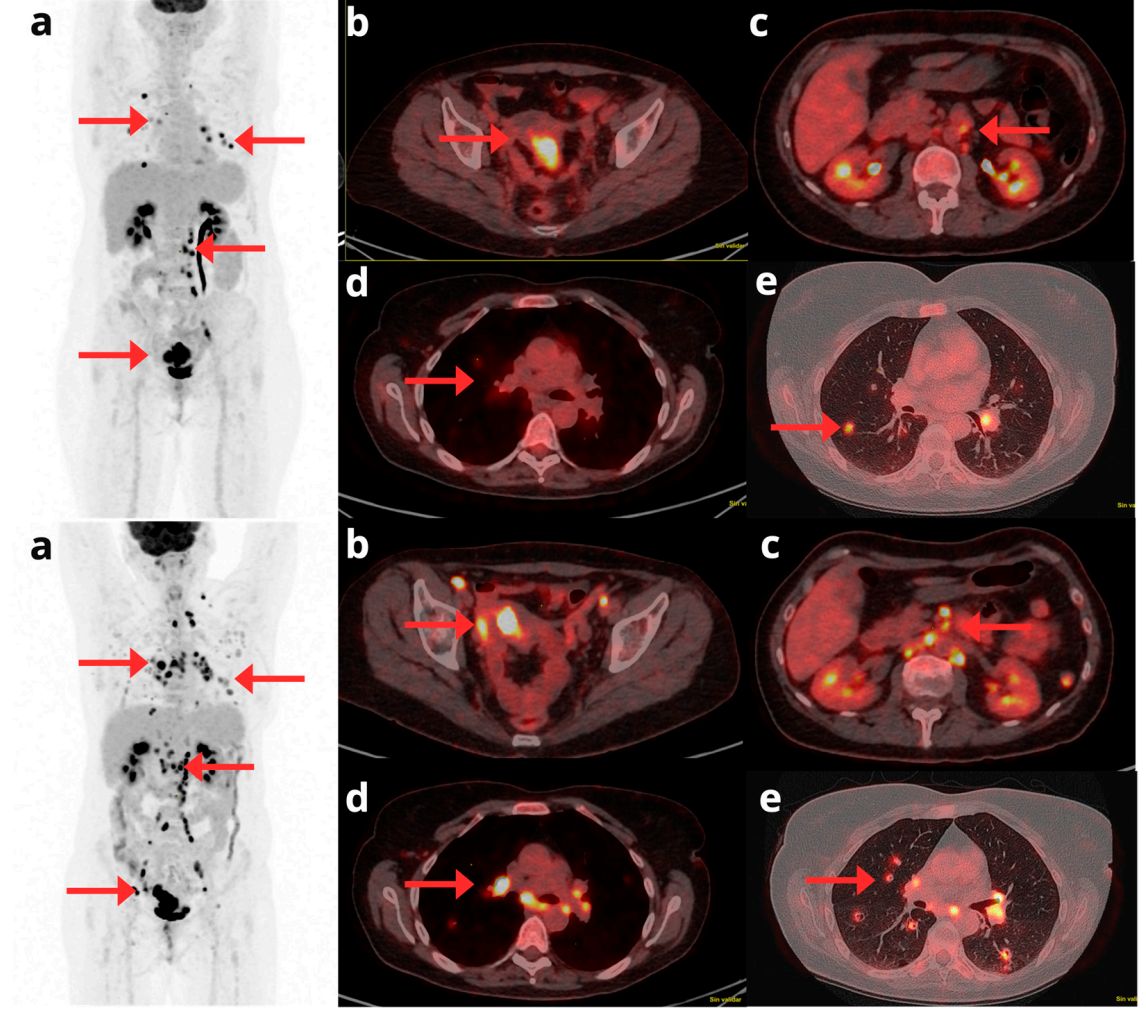
A 58-year-old woman diagnosed with stage IV clear cell EC, undergoing treatment with Pembrolizumab and Lenvatinib. A comparison is presented between the pre-treatment [^18^F]F-FDG PET/CT (**top row**) and follow-up study (**bottom row**). The maximum intensity projection PET images (**a**) provide a global view confirming clear disease progression. This progression is evident through both an increase in size and metabolism of known lesions and the appearance of multiple new metastatic foci (red arrows). Axial slices detail these findings, showing increased pelvic involvement (**b**), enlarged supra and infradiaphragmatic lymphadenopathies (**c**,**d**), and the appearance of new bilateral pulmonary nodules (**e**).

[^18^F]FDG-PET/CT plays a critical role in assessing response to systemic therapy in patients with unresectable or metastatic EC [44]. Its incorporation into post-treatment surveillance protocols has demonstrated utility for early identification of disease progression or treatment response when anatomical imaging is limited by post-therapeutic changes or low sensitivity [45]. Figure 4.

Thus, in selected advanced or high-risk cases, [^18^F]FDG-PET/CT provides a robust tool for evaluating systemic therapy response and informing management, especially in oligometastatic settings or when conventional imaging is inconclusive. In addition, early and significant metabolic response to therapy, as measured by [^18^F]FDG-PET/CT, has been associated with improved overall survival (OS) in patients with metastatic gynecological malignancies, including EC [40,46].

## 5. Recurrence Detection

**Figure 5:**
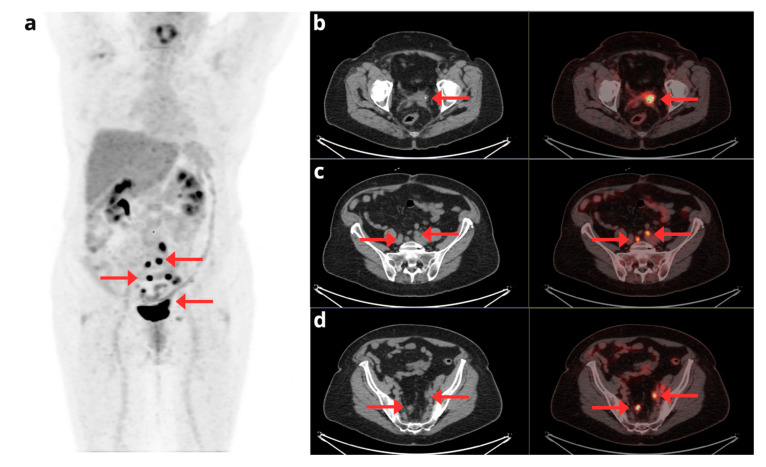
A 72-year-old woman with a history of stage IA G2 endometrioid EC, treated in October 2014 with laparoscopic hysterectomy and bilateral salpingo-oophorectomy. In May 2019, a follow-up CT scan revealed a suspicious nodular image suggestive of recurrence in the vaginal cuff, prompting a [^18^F]F-FDG PET/CT request in June 2019. The [^18^F]F-FDG PET/CT confirmed disease recurrence; the maximum intensity projection PET image (**a**) shows focal involvement in the vaginal cuff and pelvic lymph nodes (red arrows). Axial CT and PET/CT slices (**b**–**d**) localize the recurrence to the left-sided vaginal cuff (**b**) and additionally demonstrate metastatic lymphadenopathies in the common iliac chains (**c**) and bilateral internal iliac chains (**d**).

Although the overall treatment effect of EC is good, up to 15–20% of patients recur within the first 2 years after treatment. This percentage may be even higher in patients with aggressive histologic subtypes (e.g., serous or clear cell carcinoma) [40,47]. EC tends to recur in the pelvis, especially in the vaginal vault (rate of 42%) and pelvic lymph nodes, followed by para-aortic lymph nodes [48]. Reported 5-year cure rates in patients with isolated vaginal apex recurrence range from 40% to 60%, underscoring the importance of early detection through functional imaging modalities such as [^18^F]FDG-PET/CT for guiding rescue therapies [44]. Figure 5.

**Figure 6:**
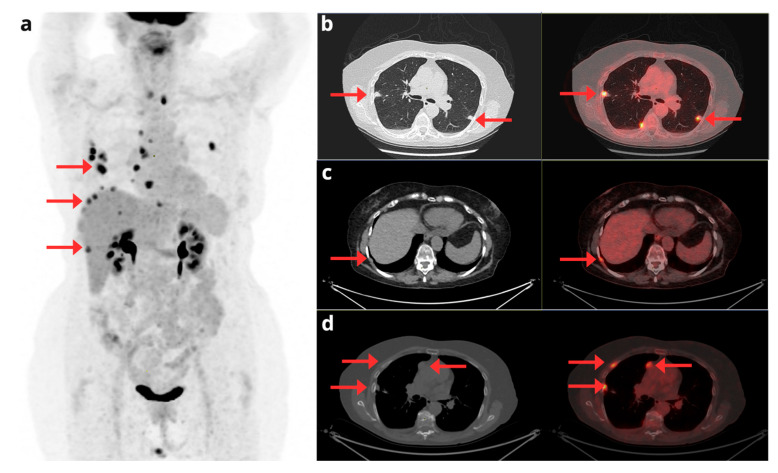
A 75-year-old woman with a history of stage IB FIGO clear cell EC, treated in April 2022 with total hysterectomy, bilateral salpingo-oophorectomy, pelvic and aortocaval lymphadenectomy, and omentectomy, followed by adjuvant chemoradiotherapy and brachytherapy. Due to the appearance of pulmonary nodules on a follow-up CT scan, a [^18^F]F-FDG PET/CT was requested. The study revealed distant disease recurrence, with the maximum intensity projection PET image (**a**) demonstrating multiple pulmonary nodules, pleural implants, and bone involvement (red arrows). Axial CT and PET/CT slices (**b**–**d**) confirmed foci of pathological hypermetabolism corresponding to bilateral pulmonary nodules (**b**), an implant in the right costal pleura (**c**), a mediastinal lymphadenopathy in a prevascular location, a subpleural pulmonary nodule in the right lower lobe, and the fourth right anterior costal arch (**d**).

Extrapelvic recurrence commonly involves the peritoneum and lungs, followed by extra-abdominal lymph nodes, liver, adrenals, brain, bones, and soft tissue [49]. Figure 6.

A meta-analysis of 11 studies, including 541 patients, confirmed the high diagnostic accuracy of [^18^F]FDG-PET/CT for detecting EC recurrence, with pooled sensitivity and specificity of 95.8% and 92.5%, respectively. Notably, [^18^F]FDG-PET/CT findings altered clinical management in 22–35% of cases [50].

**Figure 7:**
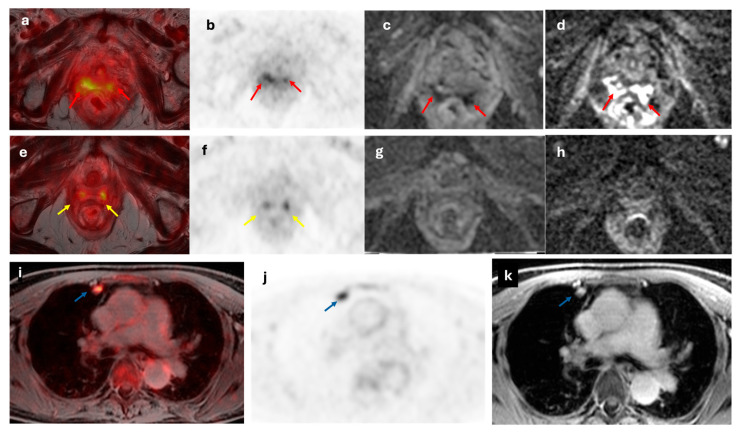
A 64-year-old woman with recurrent EC at vaginal stump (red arrow). The lesion demonstrated increased metabolic activity on axial fused [^18^F]F-FDG PET/MRI and axial [^18^F]F-FDG PET images (**a**,**b**), along with diffusion restriction on axial ADC and DWI images (**c**,**d**). The lateral vaginal walls show diffuse increased metabolic activity (yellow arrows; (**e**,**f**)) with no diffusion restriction (**g**,**h**) consistent with inflammatory changes secondary to radiotherapy. Axial fused [^18^F]F-FDG PET/MRI, axial [^18^F]F-FDG PET and axial T2-weighted MRI images (**i**–**k**, respectively) show a hypermetabolic nodule in the right upper lung lobe (blue arrow), suggestive of malignancy.

MRI provides superior soft tissue contrast, which is particularly useful for assessing local recurrences in the pelvis, lymph nodes, and peritoneum appearing as an intermediate or heterogeneous T2 signal intensity mass, with associated restricted diffusion and enhancement [37]. The evidence supports that [^18^F]FDG-PET/MRI is equivalent or outperforms [^18^F]FDG-PET/CT in recurrence detection [51,52,53]. Chao et al. [54] reported that the combination [^18^F]FDG-PET with MRI/CT significantly outperformed MRI/CT alone for overall lesion detection (AUC 0.949 vs. 0.872, respectively; *p* = 0.004), including both pelvic and extrapelvic metastases. Using [^18^F]FDG-PET/MRI, Sawicki et al. [55] identified a higher number of cancer recurrences compared to MRI alone (100% vs. 83.6%, respectively; *p* < 0.01). [^18^F]FDG-PET/MRI also offers the advantage of distinguishing post-therapeutic tissue changes from true local relapse and detecting small lymph node metastases that may appear morphologically benign on conventional imaging. Figure 7. Moreover, [^18^F]FDG-PET/MRI has demonstrated superior performance in restaging non-osteoblastic bone lesions and peritoneal and liver metastases compared to PET/CT [19].

Current guidelines recommend [^18^F]FDG-PET/CT when recurrence is suspected [3,21,22]. In follow-up, although intensive surveillance is not generally recommended, an individualized scheme based on symptomatology and clinical aspects is proposed, with periodic physical examinations and transvaginal ultrasounds. Additional imaging (whole-body CT, PET/CT, or MRI) is considered useful in the presence of suspicious findings of recurrence or in patients with stage III–IV disease, suggesting in the latter scenario a whole-body CT every 6 months for the first 3 years, and then every 6–12 months for an additional 2 years to detect asymptomatic recurrences [29,56].

**Figure 8:**
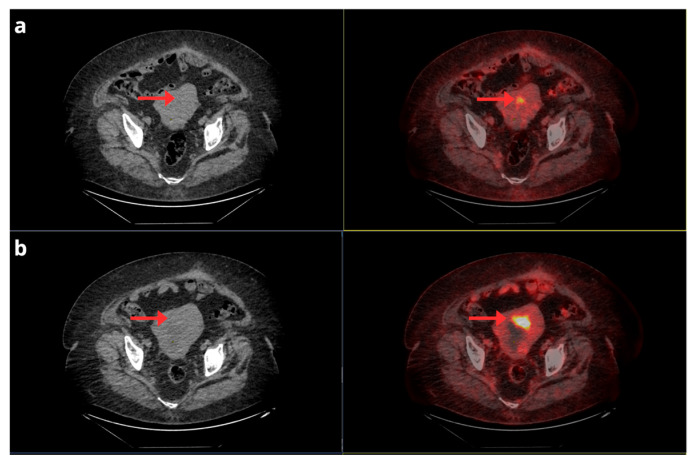
A 65-year-old female with a history of stage IB FIGO serous EC, treated with radiotherapy and brachytherapy. A comparison of two [^18^F]F-FDG PET/CT studies is presented. The axial fused CT and PET/CT slices in the top row correspond to the first post-treatment study, showing residual focal uptake in the anterior uterine wall (**a**). The axial fused CT and PET/CT slices in the bottom row, from the follow-up study 6 months later, demonstrate an increase in both the intensity and extent of this uptake suggestive of progression (**b**).

[^18^F]FDG-PET/CT should not be used for standard surveillance after treatment. However, some authors reported that this technique was able to detect recurrence in up to 12% of asymptomatic patients, influencing treatment decisions in 21.9% of cases [57,58]. Thus, although routine use of [^18^F]FDG-PET/CT in unselected EC populations is unlikely to be cost-effective, prospective studies focusing on well-defined high-risk groups are warranted to better define its potential role in personalized post-treatment surveillance strategies. Figure 8.

## 6. Radiomics: Molecular Imaging Biomarkers in Clinicopathologic and Prognosis Prediction

Radiomics is an evolving field that enables high-throughput extraction of quantitative features from medical images, such as [^18^F]FDG-PET/CT, transforming them into structured, analyzable data for predictive modelling and clinical decision support. In EC, radiomics offers a non-invasive approach to characterize tumor biology, and when integrated with clinicopathologic data and multi-omics frameworks, radiomic analysis may enhance risk stratification, treatment response prediction, and personalized prognosis [59].

### 6.1. Clinicopathological Association of ^18^F-FDG PET/CT-Derived Parameters

Several studies have demonstrated that maximum standardized uptake value (SUVmax) and the MTV of the primary tumor, using [^18^F]FDG-PET/CT, can provide indirect prognostic information, reflecting the biological aggressiveness of the tumor. SUVmax, SUVmean, MTV, and TLG have been linked with adverse features including depth of MI, lymphovascular invasion (LVI), larger tumor diameter, cervical stromal involvement, and lymph node metastases [12,60,61]. For example, Sudo et al. [62] reported an SUVmax threshold of 16 to predict LVI in patients with MI, with an accuracy of 88.2%. Thus, these parameters offer insights into tumor aggressiveness and metabolic burden. An overview of the principal quantitative parameters derived from [^18^F]FDG-PET/CT, used as imaging biomarkers, is summarized in Table 3.

According to parameters indicative of metabolic tumor burden, some groups suggested that MTV and TLG might be promising markers for LN involvement [63]. Erdogan et al. [64] proposed MTV cut-offs of 19.6, 14.3, and 29.7 mL for early FIGO, MI, and lymph node metastasis, respectively. Mapelli et al. [65] found TLG values at 40–60% thresholds to correlate with both FIGO and pathological tumor stage, and MTV60 ≥ 7.8 mL and TLG40 ≥ 77.6 showed high specificity for advanced disease.

Regarding risk stratification, [^18^F]FDG-PET/CT might serve as a predictive tool for p53 overexpression [66]. Several studies have defined SUVmax cut-offs for this purpose, and found higher values in high-risk patients compared to low-risk ones, with sensitivities and specificities moderate to high [12]. Lee et al. [67] reported a cut-off of 8.7, achieving 75.6% sensitivity, 89.5% specificity, and 81.7% accuracy. Özgü et al. [68] proposed a threshold of 6.7, with sensitivity as high as 92.9% for identifying low-risk patients. However, overlapping values between high- and low-risk groups limit the reliability of SUVmax alone. Volumetric PET parameters, such as MTV and TLG, have demonstrated greater potential for preoperative risk stratification, although TLG seems to be the most robust outcome predictor [62,65,69].

For [^18^F]FDG-PET/MRI, Shih et al. [70] reported an association of ADCmin and SUVmax with tumor grade, stage, deep MI, cervical invasion, lymphovascular space involvement, and lymph node metastasis in 69 patients with newly diagnosed EC.

### 6.2. Prognostic Value of ^18^F-FDG PET/CT-Derived Parameters

SUVmax of the primary tumor has been associated with OS, disease-free survival (DFS), and progression-free survival (PFS). Previous authors have suggested that preoperative SUVmax could act as an independent prognostic marker for recurrence and mortality in EC [13,71]. Fasmer et al. demonstrated that a high MTV can predict aggressive disease in EC [13]. Nakamura et al. [71] reported that high SUVmax independently predicted poorer DFS and OS in a cohort of 131 patients. However, other studies reported weak associations or no significant relationship with prognosis [72,73,74].

Volumetric parameters such as MTV and TLG are emerging as stronger prognostic indicators. Shim et al. [75] reported that lower MTV (HR = 1.010; 95% CI: 1.002–1.018; *p* = 0.010) and lower TLG (HR = 1.001; 95% CI: 1.000–1.002; *p* = 0.024) were significantly associated with prolonged DFS. Liu et al. [72] demonstrated that MTV > 450 mL and TLG > 2700 were associated with adverse outcomes in FIGO stage IV EC.

Using [^18^F]FDG-PET/MRI, Shih et al. [70] showed that derived biomarkers, particularly MTV and TLG, were associated with PFS and OS in patients with endometrial cancer, and MTV was an independent predictor of PFS.

Metabolic activity in lymph nodes, particularly nodal SUV (SUVN), has also demonstrated prognostic relevance. Kim et al. [76] reported that both tumor SUV (SUVT) and SUVN predicted OS, with higher SUVN linked to poorer outcomes. In line with these findings, Chung et al. [77] proposed the SUVN/SUVT ratio as a novel marker, identifying a cut-off that predicted recurrence with a moderate sensitivity and specificity.

A recent systematic review by Noriega-Alvarez et al. [78], analyzing 26 studies involving 1918 patients, supported the utility of preoperative SUVmax as a non-invasive prognostic marker of recurrence and survival in EC. Both MTV and TLG also showed potential as independent predictors of outcome, appearing more accurate than SUVmax alone for risk stratification. However, the variability in cut-off values likely reflects methodological differences between studies, including variations in PET thresholding techniques, FIGO stage distributions, and inconsistent definitions of high- and low-risk categories. Thus, further validation through large, multicenter prospective studies with sufficient follow-up is essential to validate their clinical utility.

In addition, some limitations regarding technical issues may interfere with accurate quantification of metabolic parameters, such as partial volume effects in small or necrotic lesions or physiologic [^18^F]FDG activity in the bladder and ureters. Advanced radiomic approaches, including voxel-by-voxel tumor analysis, may improve reproducibility and capture intratumoral heterogeneity more effectively. However, despite these limitations, the consistent association of MTV and TLG with prognosis across studies supports their potential as robust imaging biomarkers.

In summary, preoperative [^18^F]FDG-PET/CT FDG-PET/CT, particularly the SUVmax of the primary tumor, may provide valuable clinical and prognostic information in EC, including associations with MI histological grade, lymph node metastasis, and overall risk classification. SUVmax has been proposed as a feasible non-invasive biomarker for risk assessment and treatment planning, with potential utility as an independent prognostic indicator for recurrence and mortality. Moreover, preoperative MTV and TLG have shown promise as independent prognostic factors for predicting recurrence in EC. These volumetric parameters are believed to better reflect the overall hypermetabolic tumor burden compared to SUVmax, supporting their potential role in risk stratification.

## 7. Future Directions

### 7.1. Non-FDG Radiotracers

The compound 16a-18F-fluoro17b-estradiol ([^18^F]-FES) binds to estrogen receptor alpha (ERa), enabling a noninvasive assessment of in vivo ERa status across the whole tumor [79,80,81]. Some studies have reported the prognostic value of ERa expression in EC, with a higher level of ERa being identified as a predictive factor for favorable survival [82,83,84]. These data suggest that [^18^F]-FES PET/CT might be a prognostic biomarker [85].

In addition, a combined use of [^18^F]FDG-PET/CT and [^18^F]-FES PET/CT seems to discriminate between low- and high-grade EC, the latter showing significantly greater [^18^F]-FDG/[^18^F]-FES ratios than low-risk carcinomas or hyperplasia. Thus, this index may serve as a promising PET biomarker for distinguishing histological subtypes and prognosis [86,87]. Previous authors described that [^18^F]-FDG/[^18^F]-FES tumor uptake ratio also correlated well with PFS and OS in more aggressive EC, such as uterine sarcomas [88,89]. On the other hand, [^18^F]-FES PET/CT shows slow blood clearance and rapid metabolization that increases nonspecific signals, reducing tumor detectability [90,91]. As an alternative, 4-fluoro-11b-methoxy-16a-18 F-fluoroestradiol ([^18^F]-4FMFES) has been developed, showing a resistance to hepatic metabolism in humans [92,93]. Preliminary reports indicate that the tumor-to-background ratio is significantly higher for [^18^F]-4FMFES than for [^18^F]FDG-PET/CT in EC [94].

The [68Ga]-labeled fibroblast activation protein inhibitors ([68Ga]-FAPI) are novel PET radiopharmaceuticals that target FAPs. Cancer-associated fibroblasts demonstrate FAP expression, unlike the normal fibroblasts from which they differentiate [95]. FAP expression has been reported in various types of cancers, including gynecologic malignancies [96,97].

The utility of [68Ga]-FAPI PET/CT in EC is limited by physiological uptake in normal uterine tissue [98]. However, in special subtypes, such as clear cell EC, [68Ga]-FAPI-04 PET/CT outperforms [^18^F]FDG because of its poor uptake [99,100].

The compound 3 0-Deoxy-3′-[^18^F]fluorothymidine PET has been reported as a valuable diagnostic tool for differentiating uterine leiomyosarcoma from leiomyoma, although the experience is limited [101].

### 7.2. Machine Learning

The use of machine learning (ML) and deep learning (DL) models in preoperatively predicting several features of EC aggressiveness is a developing issue.

Regarding DL, convolutional neural networks for extracting histologic features can support pathologists in classifying the pathologic grades of gynecologic tumors [102]. However, the combination of data extracted for multiple modalities seem the most robust option [103]. In a retrospective study, 123 EC patients who underwent [^18^F]FDG-PET/CT for preoperative staging were included. Several PET variables (SUVmax, SUVmean, MTV, and TLG) and histological characteristics (histotype, MI, risk group, lymph-nodal involvement, and p53 expression) were computed on the primary tumor. ML-based classification using conventional [^18^F]FDG-PET/CT parameters and clinical data demonstrated the ability to characterize the investigated features of EC aggressiveness, providing a non-invasive way to support preoperative stratification of EC patients [104].

Summarizing, based on the limited standardization and interpretation of the great amount of managed data, the information derived from a single modality might not be consistent and sufficient to capture the heterogeneity of oncological processes. Thus, to advance toward a precision oncology, the use of multimodal data models seems the future direction.

## 8. Conclusions

The reduced spatial resolution limits the diagnostic performance of [^18^F]FDG-PET/CT in the primary tumor assessment, especially in early-stage disease. However, [^18^F]FDG-PET/CT exhibits greater reliability than conventional imaging modalities in the diagnosis of lymph node metastases and distant metastases.

The functional imaging capability of [^18^F]FDG-PET/CT provides important prognostic information and may guide clinical decisions regarding the continuation, modification, or escalation of therapy. In addition, although the utility of [^18^F]FDG-PET/CT in monitoring response is not yet standardized in clinical guidelines, emerging evidence suggests that it can offer valuable insights into post-therapy metabolic response.

The compound [^18^F]-FDG PET/MRI enhances the accuracy of TNM staging in EC, offering superior preoperative evaluation of tumor invasion, lymph node involvement, and distant spread, with respect to the rest of imaging techniques, which supports better risk stratification and surgical planning. This modality is especially helpful in cases with ambiguous findings from other imaging methods, and may be critical for planning fertility-preserving or minimally invasive strategies.

The evidence strengthens the clinical relevance of [^18^F]FDG-PET-based metabolic parameters for predicting tumor biology, treatment outcomes, and prognosis in EC. The incorporation of these molecular imaging biomarkers into risk stratification multimodal models holds promise for refining personalized treatment strategies and guiding clinical decision-making.

Regarding non-[^18^F]-FDG radiotracers, further research is required to evaluate their role in the management of EC.

In summary, the choice between PET/CT and PET/MRI depends on the specific clinical situation and the goals of imaging. A careful evaluation of the patient’s individual needs and preferences, along with the available resources, is essential for making the most appropriate choice.

## Figures and Tables

**Table 1:** TNM stage and description of FIGO classification of endometrial cancer.

FIGO Stage	FIGO Description	T	N	M
I	Tumor confined to the body of the uterus	T1	N0	M0
IA	No myometrial invasion or less than half	T1a	N0	M0
IB	Myometrial invasion equal to or greater than half	T1b	N0	M0
II	Tumor invading cervical stroma without extending beyond the uterus	T2	N0	M0
III	Local and/or regional extension of the tumor	T3	N0–N1	M0
IIIA	Tumor invading the serosa of the uterine body and/or adnexa	T3a	N0	M0
IIIB	Vaginal and/or parametrial involvement	T3b	N0	M0
IIIC1	Positive pelvic nodes	T1–T3	N1	M0
IIIC2	Positive aortic nodes with or without positive pelvic nodes	T1–T3	N1	M0
IVA	Tumor invading the lining of the bladder and/or rectum	T4	Any N	M0
IVB	Distant metastases, including intra-abdominal and/or inguinal node metastases	Any T	Any N	M1

T: tumor, N: lymph nodes, M: metastases.

**Table 2:** Pros and cons of PET/CT and PET/MRI in EC initial staging.

	Pros and Cons of PET/CT	Pros and Cons of PET/MRI
**T**	Pros: shorter acquisition. Cons: - Low soft tissue resolution. - Not accurate for the evaluation of MI or CI.	Pros: - Superior soft tissue contrast. - More accurate assessment of local tumor extent as MI or CI (may help guide radiation therapy planning). - Possibility of association of radiomic variables of both techniques in prognosis assessment. Cons: longer acquisition.
**N**	Pros: high specificity. Cons: low sensitivity.	Pros: high specificity (PET adds specificity to MRI). Cons: moderate-low sensitivity.
**M**	Pros: effective distant metastases global detection. More optimal in lung metastases detection. Cons: limitations in liver metastases.	Pros: more optimal in liver, soft tissues, and osteolitic bone metastases detection. Cons: longer and less comfortable study for patients.

MI: myometrial invasion; CI: cervical involvement.

**Table 3:** Quantitative ^18^F-FDG PET/CT biomarkers.

Biomarker	Description
SUV	Measure the uptake of the radioactive tracer in a specific ROI to assess the activity and metabolism of tissues: SUV = Tracer concentration in ROI (kBq/mL)/Injected dose per body weight (kBq/g)
SUVmean	Calculating the average tracer uptake in the selected ROI A comprehensive assessment of the overall tracer uptake within the ROI, useful for areas with varying tracer uptake (e.g., tumors)
SUVmax	Indicating the highest level of tracer uptake within a defined ROI
MTV	The metabolically active volume of the tumor (i.e., the portion of the tumor with a high SUV)
TLG	Provides a more comprehensive measure of tumor activity than SUVmax or SUVmean alone. TLG = SUVmean × MTV

SUV: standardized uptake value; ROI: region of interest; SUVmean: mean standardized uptake value; SUVmax: maximum standardized uptake value; MTV: metabolic tumor volume; TLG: total lesion glycolysis.

## Data Availability

All data generated or analyzed during this study are included in this published article.

## Abbreviations

The following abbreviations are used in this manuscript:

EC	Endometrial cancer
PET/CT	Positron emission tomography/computed tomography
[^18^F]FDG	Fluorine-18–2-fluoro-2-deoxy-D-glucose
SUVmax	Maximum standardized uptake value
MTV	Metabolic tumor volume
TLG	Total lesion glycolysis
MRI	Magnetic resonance imaging
PET/MRI	Positron emission tomography/ magnetic resonance imaging
ESGO	European Society of Gynecological Oncology (ESGO)
ESTRO	European Society for Radiotherapy and Oncology
EANM	European Association of Nuclear Medicine
CRT	Chemoradiotherapy
SNMMI	Society of Nuclear Medicine and Molecular Imaging
[68Ga]-FAPI	[68Ga]-labeled fibroblast activation protein inhibitors
[^18^F]-4FMFES	4-fluoro-11b-methoxy-16a-18 F-fluoroestradiol
[^18^F]-FES	16a-18F-fluoro17b-estradiol
ERa	Estrogen receptor alpha
FIGO	International Federation of Gynecology and Obstetrics
OS	Overall survival
PPV	Positive predictive value
MI	Myometrial invasion
G	Grade
T	Tumor
N	Lymph node
M	Distant metastases
ML	Machine learning
DL	Deep learning
